# ETV5 Regulates Sertoli Cell Chemokines Involved in Mouse Stem/Progenitor Spermatogonia Maintenance

**DOI:** 10.1002/stem.508

**Published:** 2010-10-26

**Authors:** Liz Simon, Gail C Ekman, Thomas Garcia, Kay Carnes, Zhen Zhang, Theresa Murphy, Kenneth M Murphy, Rex A Hess, Paul S Cooke, Marie–Claude Hofmann

**Affiliations:** a Department of Veterinary Biosciences, University of IllinoisUrbana, Illinois, USA; b Department of Pathology and Immunology, Washington University School of MedicineSt. Louis, Missouri, USA; c Howard Hughes Medical InstituteUSA; d Divison of Nutritional Sciences and University of IllinoisUrbana, Illinois, USA; e Divison of Nutritional Sciences and University of IllinoisUrbana, Illinois, USA

**Keywords:** *Ets*-variant gene 5, testis, spermatogonial stem cells, Sertoli cells, chemokines

## Abstract

Spermatogonial stem cells are the only stem cells in the body that transmit genetic information to offspring. Although growth factors responsible for self–renewal of these cells are known, the factors and mechanisms that attract and physically maintain these cells within their microenvironment are poorly understood. Mice with targeted disruption of Ets variant gene 5 (*Etv5*) show total loss of stem/progenitor spermatogonia following the first wave of spermatogenesis, resulting in a Sertoli cell–only phenotype and aspermia. Microarray analysis of primary Sertoli cells from *Etv5* knockout (*Etv5^−/−^*) versus wild–type (WT) mice revealed significant decreases in expression of several chemokines. Chemotaxis assays demonstrated that migration of stem/progenitor spermatogonia toward *Etv5*^−/−^ Sertoli cells was significantly decreased compared to migration toward WT Sertoli cells. Interestingly, differentiating spermatogonia, spermatocytes, and round spermatids were not chemoattracted by WT Sertoli cells, whereas stem/progenitor spermatogonia showed a high and significant chemotactic index. Rescue assays using recombinant chemokines indicated that C-C-motif ligand 9 (CCL9) facilitates Sertoli cell chemoattraction of stem/progenitor spermatogonia, which express C-C-receptor type 1 (CCR1). In addition, there is protein–DNA interaction between ETV5 and *Ccl9*, suggesting that ETV5 might be a direct regulator of *Ccl9* expression. Taken together, our data show for the first time that Sertoli cells are chemoattractive for stem/progenitor spermatogonia, and that production of specific chemokines is regulated by ETV5. Therefore, changes in chemokine production and consequent decreases in chemoattraction by *Etv5^−/−^* Sertoli cells helps to explain stem/progenitor spermatogonia loss in *Etv5^−/−^* mice.

## INTRODUCTION

In the mammalian testis, spermatogonial stem cells (SSCs) form a small subpopulation of germ cells that self-renew to maintain sperm production throughout life. SSCs reside in the basal part of the epithelium lining the seminiferous tubules and are in close association with Sertoli cells that provide germ cells with growth and differentiation factors. Despite recent progress in characterizing SSCs, their true identity has not been demonstrated, and transplantation assays have indicated that all early type A spermatogonia (A_single_, A_paired_, and A_aligned_) might have stem cell potential [1, 2]. A_aligned_ spermatogonia further develop into differentiating spermatogonia (A_1_-A_4_), type B spermatogonia and spermatocytes that will undergo meiosis, become haploid spermatids and ultimately sperm [3]. Of particular interest is the perinatal differentiation of gonocytes and SSCs into more mature germ cells, which establishes the first and subsequent waves of spermatogenesis. This period of testicular development is associated with considerable structural changes in the seminiferous epithelium, during which the size of the testis increases over 80-fold in the mouse. This expansion is due to the fact that the somatic Sertoli cells that support germ cells proliferate extensively until day 12–16 after birth [4]. It has been postulated that this early time period, during which there is simultaneous proliferation of both Sertoli cells and spermatogonia, has regulatory requirements that are unique [5]. With each Sertoli cell division a new potential stem cell niche is created, which would be filled by the continued proliferation of stem/progenitor spermatogonia followed by lateral migration of these cells along the basement membrane [5]. Thereafter, the cyclic process of spermatogenesis initiated from SSCs nested within their niche during the first wave is maintained throughout adult life.

The niche represents the microenvironment of a stem cell within a specific tissue that regulates its fate, ensuring maintenance or homeostasis between self-renewal and differentiation. In the mammalian testis, the SSC niche is mainly defined by physical associations with Sertoli cells and the basement membrane to which SSCs adhere [6, 7]. In addition, the vascular network [8] and interstitial cells found between seminiferous tubules communicate with SSCs and are critical for their spatial localization along the basement membrane. Sertoli cells produce growth factors that are essential for SSC maintenance. In particular, they secrete glial cell line-derived neurotrophic factor (GDNF), which plays a major role in promoting SSC self-renewal in vivo [9, 10] and in vitro [11–13]. GDNF secreted by Sertoli cells binds to a receptor complex formed by association of the receptors GFRa1 (GDNF family membrane receptor alpha 1) and RET (Rearranged during transfection), which brings about the intracellular response. In the testis, GFRa1 is expressed by A_single_ and possibly A_paired_ and A_aligned_ spermatogonia [11, 14, 15]. Hence, GFRa1 was selected to enrich stem/progenitor spermatogonia after STAPUT isolation of type A spermatogonia in our experiments. Sertoli cells also produce factors like basic fibroblast growth factor and epidermal growth factor, which are essential for stem cell maintenance [7, 12] and have stimulatory effects on Ets variant gene 5 (*Etv5*) expression [16].

The transcription factor ETV5 (also known as *Ets*-variant gene 5) is a protein that is critical for maintenance of stem/progenitor spermatogonia within their niche [17]. ETV5 is expressed by both Sertoli and germ cells, but its precise role in each cell type remains to be established [17–19]. Mice with a global deletion of *Etv5* (*Etv5^-/-^*) have a first wave of spermatogenesis, but there is complete loss of stem/progenitor spermatogonia by day 36 [20], resulting in a Sertoli cell-only phenotype and ultimately aspermia in adulthood [17]. Loss of ETV5 in stem/progenitor spermatogonia induces a decrease in their self-renewal in vivo and in vitro [19, 21]. However, this does not account for the total loss of these cells. Over the years, it has become evident that the developmental fate of a stem cell is not only determined by intrinsic factors, but is also influenced by the availability and quality of stem cell niches [1]. Therefore, a critical question for understanding the role of ETV5 in spermatogenesis is to determine its function in Sertoli cells.

Chemokines are small secreted cytokines that were initially found to play a major role in leukocyte migration and communication [22, 23]. However, there is growing evidence suggesting a role of chemokines in homing and maintenance of stem cells in their niche [24, 25]. In the embryo, primordial germ cell (PGC) migration is mediated by the chemokine C-X-C motif ligand 12 (CXCL12), also known as stromal cell-derived factor-1 (SDF-1) [26]. However, little is known about the role of chemokines in mediating communication between Sertoli cells and germ cells after birth and maintaining stem/progenitor spermatogonia in their microenvironment.

Microarray analysis of *Etv5^-/-^* Sertoli cells revealed a decrease in several chemokines-encoding genes such as C-C-motif ligand 9 (*Ccl9*), *Ccl7*, *Cxcl5*, and *Cxcl12* [17]. As a role of chemokines in stem cell homing has been proposed in other systems [24–26], we hypothesized that ETV5 in Sertoli cells might be regulating chemokine expression, and that these proteins could directly or indirectly be involved in migration and/or retention of stem/progenitor spermatogonia in their microenvironment. Our data indicate that there is a decrease in chemoattraction of stem/progenitor spermatogonia toward Sertoli cells following loss of ETV5. This, together with a decrease in the rate of proliferation [19, 21], explains the ultimate loss of these cells in *Etv5^-/-^* mice. One of these chemokines, CCL9, is strongly expressed by Sertoli cells, and we have localized its receptor, CCR1, to undifferentiated spermatogonia. Further, we show possible protein-DNA interaction between ETV5 protein and the *Ccl9* promoter. These data indicate that chemokine signaling is involved in the migration of stem/progenitor spermatogonia to newly established Sertoli cells.

## MATERIALS AND METHODS

### Animals

C57Bl/6 dams with male pups (5- to 6-day-old) were obtained from Charles River (Boston, MA, http://www.criver.com). *Etv5^-/-^* (germline knockout of exons 2–5) [17] and C57Bl/6 mice were bred and maintained in the animal facility at University of Illinois. Mice were housed at 25°C with a 12L:12D photoperiod and given water and a standard rodent chow diet. All animal experiments were approved by the IACUC at the University of Illinois and conducted in accordance with the National Institute of Health Guide for the Care and Use of Laboratory Animals.

### Isolation of Stem/Progenitor, Type B Spermatogonia, Leptotene/Zygotene, Pachytene Spermatocytes, and Round Spermatids

Germ cells were isolated by the STAPUT method, which utilizes gravity sedimentation on a 2%–4% bovine serum albumin (BSA) gradient [27, 28]. Type A spermatogonia (Type A) were isolated from C57Bl/6 pups at days 5–6 (60–80 pups). Type B spermatogonia and leptotene/zygotene spermatocytes (Type B, L/Z) were isolated from C57Bl/6 mice at day 12 (10–12 pups), pachytene spermatocytes (P) at day 21 (5–8 mice), and round spermatids (RS) at day 45 (3–4 mice). Briefly, testes were decapsulated and subjected to a two-step enzymatic digestion protocol, to eliminate peritubular cells and Leydig cells [27–29]. The resulting single cell suspension, containing Sertoli cells and germ cells, was washed by centrifugation and resuspended in 0.5% BSA in Dulbecco's modified Eagle's medium (DMEM)/F12. Cells were separated based on gravity sedimentation and the different fractions were collected using a fraction collector (Bio-Rad, Hercules, CA, http://www.bio-rad.com). The germ cells were identified by size and morphological characteristics using a light microscope. The size of cells were ∼14–16 µm, 8 µm, 12–18 µm [27], and 10–11 µm [30] for Type A, Type B and L/Z, P, and RS, respectively. The cell fractions that were enriched for the respective cell types were pooled, counted, and resuspended in DMEM/F-12 supplemented with 10% synthetic Nu-Serum (BD Biosciences, San Jose, CA, http://www.bdbiosciences.com), penicillin-streptomycin (1 ml/100 ml; Invitrogen, Carlsbad, CA, http://www.Invitrogen.com) for chemotaxis assays. All enzymes were purchased from Sigma (St. Louis, MO, http://www.sigmaaldrich.com). For isolation of stem/progenitor spermatogonia, the type A cell suspension was plated on lectin-coated plates for up to 30 minutes (differential plating) to remove contaminating Sertoli or peritubular cells [31]. The supernatant containing Type A spermatogonia (95%–98% purity, 4 × 10^6^ cells for 60 pups, 6-day-old) was resuspended in DMEM/F-12 with 10% Nu-Serum for enrichment of stem-progenitor spermatogonia with magnetic-activated cell sorting (MACS) using GFRa1 as the selecting marker [32].

### Magnetic-Activated Cell Sorting

The enriched Type A spermatogonial fraction was incubated with a GFRa1 antibody (SC-10716, Santa Cruz Biotechnology, Santa Cruz, CA, http://www.scbt.com) at a concentration of 5 µg/10^6^ cells overnight at 4°C with gentle rocking. The GFRa1^+^ fraction was isolated using Miltenyi MicroBeads (Miltenyi Biotec, Auburn, CA, http://www.miltenyibiotec.com) [32], and resulted in a germ cell population (75,000–100,000 cells for 60 pups, 6-day-old) containing 70%–80% stem-progenitor spermatogonia. For chemotaxis assays, ∼300 pups were needed/experiment. The same procedure was followed for isolating c-KIT^+^ cells (differentiating spermatogonia) from GFRa1^-^ spermatogonia using anti-c-KIT as primary antibody (eBioscience, San Diego, CA, http://www.ebioscience.com, 14-1172, rat monoclonal) and anti-rat IgG MicroBeads.

### Sertoli Cell Isolation and Culture

Sertoli cells were isolated from 5-day-old *Etv5^-/-^* or wild-type (WT) pups according to standard methods widely used by others and our laboratories [4, 33–36]. Briefly, testes were decapsulated and digested using a 2-step enzymatic digestion protocol, to eliminate peritubular and Leydig cells. The resulting cell suspension, containing Sertoli cells and spermatogonia, was trypsinized to eliminate clumps, resuspended in serum-free DMEM/F-12 media (minimal media), and plated on matrigel-coated plates. The media was changed after 4 hours to remove spermatogonia, and the Sertoli cells were cultured for 48 hours. This method of isolation yields a highly homogenous Sertoli cell population with a purity of >95%. Lack of serum averts the proliferation of any residual peritubular cells. Although contamination by peritubular cells is unavoidable, no germ cells and only <1% Leydig cells can be detected. This method also preserves the functionality of Sertoli cells for at least 7 days, such as response to hormones and growth factors [33, 34, 36–39].

### Chemotaxis Assays

Chemotaxis assays were performed using 8-µm-pore Cytoselect 24-well assay plates (colorimetric format; Cell Biolabs, San Diego, CA, http://www.CellBiolabs.com). Sertoli cells isolated from 5-day-old *Etv5^-/-^* and WT testes were seeded at a concentration of 2.5 × 10^5^ cells in the lower chambers of the transwell units and cultured in minimal media for 48 hours prior to migration assays. WT stem/progenitor spermatogonia or other germ cells were seeded in minimal media into the upper chamber at a concentration of 5 × 10^5^ cells/insert. The cells were allowed to migrate for 24 hours at 34°C, 5% CO_2_, and maximum humidity. DMEM/F12 with 10% fetal bovine serum (FBS) was used as a positive control in the lower chamber in all experiments, and minimal media alone, without any added cells, chemokines or serum, was used as a negative control. After 24 hours of incubation, the cells that migrated through pores in the membrane were fixed, stained, and lysed. The optical density of the lysed solution was measured at 560 nm and the chemotactic index was calculated. The chemotactic index is the optical density measurement of stem/progenitor spermatogonia that migrated toward the chemoattractant in the lower chamber compared with the negative control (minimal media or *Etv5^-/-^* Sertoli cells).

Initially a dose-response of recombinant proteins CCL9, CCL12, and CXCL5 to attract C18-4 cells was performed. C18-4 is an immortalized SSC cell line that expresses many SSC markers such as GFRa1, OCT-4, and deleted in azoospermia-like (DAZL) [40, 41]. Chemokines were added to DMEM/F-12 in the lower chamber of the transwell unit and incubated with C18-4 cells in the upper chamber for 24 hours. The optimum concentration was standardized as 50 ng/ml for all proteins individually (not shown).

### RNA Extraction and Real-Time Polymerase Chain Reaction

RNA was extracted from isolated and cultured Sertoli and germ cells using the RNeasy mini or micro kit according to manufacturer's instructions (Qiagen, Valencia, CA, http://www.qiagen.com). The samples were treated with DNase to prevent DNA contamination. First-strand cDNA was synthesized from total RNA (1 or 0.5 µg) using Superscript reverse transcriptase III and random primers (Invitrogen, Carlsbad, CA, http://www.Invitrogen.com). The expression value of each gene was normalized to the amount of an internal control gene (18S ribosomal RNA) to calculate a relative amount of RNA in each sample. The expression value of each gene in WT or mock siRNA controls was arbitrarily defined as 1 unit. Real-time polymerase chain reaction (PCR) assays were carried out in triplicate (three technical × three biological repeats) and the normalized expression values for all WT, *Etv5^-/-^* Sertoli samples, and germ cells averaged. A relative quantitative fold change was determined using the ΔΔ Ct method. ABI Taqman gene expression assays used for specific transcripts were: Mm 01617100_m1 (*Ccl12*), Mm 00443113_m1 (*Ccl7*), Mm 00441260_m1 (*Ccl9*), Mm 00436451_g1 (*Cxcl5*), Mm 01216147_m1 (*Ccr1*), Mm00436304_m1 (*Ret*) and Hs 9999901_s1 (18S) (Applied Biosystems, Carlsbad, CA, http://www.appliedbiosystems.com).

### Superarray Gene Analysis

RNA was extracted from isolated Sertoli cells using the RNeasy mini kit (Qiagen, Valencia, CA, http://www.qiagen.com). Template cDNAs were prepared from total RNA (1 µg) of *Etv5^-/-^* and WT Sertoli cells (RT^2^ PCR array first strand kit, SABiosciences, Frederick, MD, http://www.sabiosciences.com) and were characterized in triplicates using the Mouse RT^2^ chemokines and receptors profiler PCR arrays (SABiosciences, Frederick, MD, http://www.sabiosciences.com) with the RT^2^ SYBR Green/Fluorescein PCR master mix on a ABI 7000 thermal cycler (Applied Biosystems, Carlsbad, CA, http://www.appliedbiosystems.com).

### Electromobility Shift Assays

Electromobility shift assays (EMSAs) were carried out using 3' biotinylated probes (Invitrogen, Carlsbad, CA, http://www.invitrogen.com) designed to include the three Ets binding sites (EBSs) found in the *Ccl9* genomic sequence (Accession number AL596122) and a chemiluminescent kit (Cat. No. 20148, Thermo Scientific, Pittsburgh, PA, http://www.thermo.com). Sense and antisense primers to the following sequences (EBS in bold) were annealed and used as probes;

1. EBS1-AATTTGAACTTT**GGAAA**GCCCCAAGTCACC,
2. EBS2-GGAGAAATCCTTCT**GGAAA**TATTTACTCTC and
3. EBS3-CTCTAGTACCAG**GGAAT**TTAACACCCTCTTC.

EBS1 and EBS2 did not produce a shift (data not shown). Mutant sequence to EBS3 used as probes was: CTCTAGTACCA**CTCGAG**TTAACACCCTCTTC. Nuclear extracts were prepared from TM4, a Sertoli cell line, transfected 48 hours with an *Etv5* expression vector [42] (pErmFLAG, a generous gift from Dr. John Shannon, University of Cincinnati) or with control vector. Nuclear extracts were incubated in binding reaction buffer (Thermo Scientific, Pittsburgh, PA, http://www.thermo.com) with 2.5% glycerol, 50 mM KCl, 2 mM MgCl_2,_ NP-40 (0.04%), 50 ng/µl polydeoxyinosine-polydeoxycytidine acid (poly dI/dC), 1 mM dithiothreitol, 2 mM EDTA, and 20 fM biotinylated (B) probe at room temperature for 20 minutes. Competitor reactions contained nonbiotinylated (NB) probes at 200-fold excess. For supershift assays, the reaction mixture was incubated with 0.5 µg of ETV5 antibody (sc-22807, Santa Cruz Biotechnology, Santa Cruz, CA, http://www.scbt.com) for an additional 20 minutes. Screening of different concentrations of ETV5 antibody and NB probe was not performed in this study. Each reaction was fractionated on a 4% nondenaturing acrylamide gel.

For producing purified ETV5 protein, a cDNA fragment containing the ETV5 binding domain was generated using PCR and inserted into the pET-41a(+) vector (Novagen/EMD Biosciences, Gibbstown, NJ, http://www.emdchemicals.com) according to manufacturer's instructions, and the sequence verified. The pET41a-ETV5 expression construct was transfected into *E. Coli* BL21 (DE3) cells. The cells were grown with 30 µg/ml kanamycin, and optimal expression was induced with 1 mM isopropyl beta-D-1-thiogalactopyranoside (IPTG). The ETV5 protein was purified using B-PER 6XHis purification kit (Thermo Scientific, Pittsburgh, PA, http://www.thermo.com) according to manufacturer's instructions. The ETV5 protein was incubated with binding buffer, B or NB probe to perform EMSAs.

### Immunohistochemistry, Immunocytochemistry and Immunofluorescence

#### Tissue sections

Testes from 6 to 8-day WT and *Etv5-/-* mice were fixed in 4% paraformaldehyde, embedded in paraffin and cut into 5 m thick sections. Slides were deparaffinized in xylene and rehydrated in decreasing ethanol gradients. The slides were boiled for 10 minutes in 0.01 M citrate buffer (pH = 6.0) for antigen retrieval, then blocked with 10% normal goat serum for 30 minutes. The primary antibodies used were anti-CCL9 (rabbit polyclonal ab9913, 1:500 dilution, Abcam, Cambridge, MA, http://www.abcam.com), anti-CCR1 (rabbit polyclonal NB100-702, 1:300 dilution, Novus Biologicals, http://www.NovusBio.com) and anti-germ cell nuclear antigen 1 (GCNA1) (rat monoclonal, 1:5 dilution, a generous gift from Dr. George Enders, University of Kansas Medical Center). For immunohistochemistry of CCR1, the secondary antibody was a biotinylated goat anti-rabbit IgG (1:500 dilution, Vector Laboratories, Burlingame, CA, http://www.vectorlabs.com). Binding of the biotinylated antibody was revealed with the immunoperoxidase technique using a 3,3'diaminobenzidine (DAB) kit (SK-4100, Vector Laboratories, Burlingame, CA, http://www.vectorlabs.com), which gives a brown precipitate at the reaction sites. Control slides were prepared by replacing the primary antibody in the staining procedure with goat serum at same dilutions. Photomicrographs were taken with a BX51 Olympus microscope (Olympus Corp., Melville, NY, Center Valley, PA, http://www.olympusamerica.com). For immunofluorescence, the secondary antibodies used were goat anti-rabbit IgG Alexa Fluor 488 and goat anti-rat IgG AlexaFluor 594 (1:300 dilution, Molecular Probes/Invitrogen, Carlsbad, CA, http://www.Invitrogen.com). Control slides were prepared as above. Slides were mounted using Vectashield with 4',6-diamidino-2-phenylindole (DAPI, H-1200, Vector Labs, Burlingame, CA, http://www.vectorlabs.com) and photomicrographs taken using an Olympus IX70 fluorescence microscope (Olympus Corp., Melville, NY, http://www.olympusamerica.com).

#### Cell smears

Cell smears of enriched stem/progenitor spermatogonia and other germ cells were prepared and fixed in 4% paraformaldehyde for immunocytochemistry or immunofluorescence. The primary antibodies used were anti-mouse VASA homolog (mVH, rabbit polyclonal sc-67185, 1:500 dilution, Santa Cruz Biotechnology, Santa Cruz, CA, http://www.scbt.com), anti-GCNA1 (rat monoclonal, 1:5 dilution, Dr. George Enders, University of Kansas Medical Center) and anti-GFRalpha1 (rabbit polyclonal sc-10716, 1:200 dilution, Santa Cruz Biotechnology, Santa Cruz, CA, http://www.scbt.com). For immunocytochemistry of mVH, the secondary antibody was a biotinylated goat anti-rabbit IgG (1:500 dilution, Vector Laboratories, Burlingame, CA, http://www.vectorlabs.com). Visualizing of the biotinylated antibody was performed as above with the immunoperoxidase technique using a DAB kit (SK-4100, Vector Laboratories, Burlingame, CA, http://www.vectorlabs.com). Control slides were prepared by replacing the primary antibody in the staining procedure with goat serum at same dilutions. Photomicrographs were taken with a BX51 Olympus microscope (Olympus Corp., Melville, NY, Center Valley, PA, http://www.olympusamerica.com). For immunofluorescence of GFRalpha-1 and GCNA1, the secondary antibodies used were goat anti-rabbit IgG Alexa Fluor 488 and goat anti-rat IgG AlexaFluor 488 respectively (1:1000 dilution, Molecular Probes/Invitrogen, Carlsbad, CA, http://www.invitrogen.com). Control slides were prepared as above. Slides were mounted using Vectashield with 4',6-diamidino-2-phenylindole (DAPI, H-1200, Vector Labs, Burlingame, CA, http://www.vectorlabs.com) and photomicrographs taken with an Olympus IX70 fluorescence microscope (Olympus Corp., Melville, NY, http://www.olympusamerica.com).

### Western Blots

Isolated seminiferous tubules from 4-, 10-, 35-, and 90-day-old WT mice were washed with cold phosphate-buffered saline (PBS) and placed on ice and lysed with RIPA buffer containing protease inhibitors (1 mM phenylmethylsulphonylfluoride, 50 µg/ml aprotinin, and 5 µg/ml leupeptins). Proteins were resolved on 4%–20% Tris-HCl gels in a Mini Protean 3 Cell (Biorad, Hercules, CA, http://www.bio-rad.com). After SDS-PAGE, gels were electroblotted at 100 V for 60 minutes onto polyvinylidene diflouride membranes using a Mini Protean 3 Cell. Blots were incubated with anti-CCL9 (rabbit polyclonal ab9913, 1:1000 dilution, Abcam, Cambridge, MA, http://www.abcam.com) and anti-Wilm's tumor antigen 1 (WT1; mouse monoclonal sc-7385, 1:1000 dilution, Santa Cruz Biotechnology, Santa Cruz, CA, http://www.scbt.com) primary antibodies. Antigen-antibody complexes were detected by incubation of membranes at room temperature with the appropriate, horseradish peroxidase-labeled anti-IgG antibodies, and developed using Supersignal West Pico Chemiluminescent substrate (Thermo Scientific, Pittsburgh, PA, http://thermo.com).

### RNA Interference

*Ccr1* downregulation: Stem/progenitor spermatogonia from WT testes were seeded into 24-well plates at a concentration of 2 × 10^4^ in 200 µl of DMEM/F-12 with 10% Nu-Serum (BD Biosciences, Franklin Lakes, NJ, http://www.bdbiosciences.com). The cells were then transfected with *Ccr1* or mock siRNAs (Invitrogen, Carlsbard, CA, http://www.invitrogen.com) using RNAiFect kit according to manufacturer's instructions (Qiagen, Valencia, CA, http://www.qiagen.com). RNA was isolated after 24 hours to assess the efficiency of gene silencing. For chemotaxis assays, stem/progenitor spermatogonia that were transfected with *Ccr1* siRNA for 24 hours were then allowed to migrate toward WT Sertoli cells for 24 hours and the chemotactic index was assessed.

### Statistical Analysis

For all experiments, including RNA interference studies, data were collected from three independent experiments, each done in triplicate. For the chemotaxis assays, data were obtained from three independent experiments, each done in duplicates or triplicates. Data were analyzed by one-way analysis of variance followed by Tukey–Kramer's multiple comparison tests. Statistical analyses were performed using Graph Pad Prism 4.0 (Graph Pad Software, Inc., San Diego, CA). Data were expressed as mean ± SEM, and differences between groups were considered significant at *p* < .05.

## RESULTS

### Expression of Specific Chemokines Is Decreased in Sertoli Cells from *Etv5^-/-^* Mice

**Figure 1:**
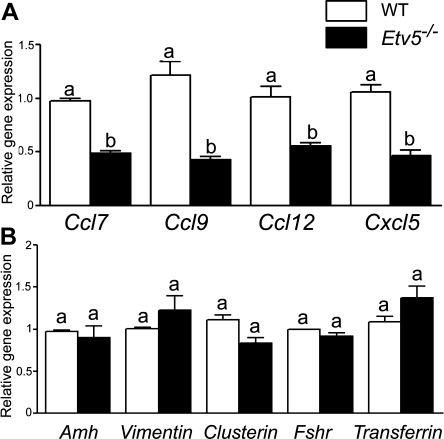
mRNA levels of chemokines are decreased in *Etv5^-/-^* Sertoli cells. (A): mRNA levels of *Ccl7*, *Ccl9*, *Ccl12*, and *Cxcl5* in freshly isolated Sertoli cells of *Etv5^-/-^* mice were decreased 50%–70% compared with age-matched WT mice as determined by real-time polymerase chain reaction. (B): mRNA expression of other Sertoli cell genes. Anti-Mullerian hormone (*Amh*), vimentin, clusterin, follicle stimulating hormone receptor (*Fshr*), and transferrin were not altered in *Etv5^-/-^* mice. Results are expressed as mean ± SEM (*n* = 3 for each genotype). Values with different letters were significantly different (*p* < .01). Abbreviations: *ETV5*, Ets variant gene; WT, wild type.

Our initial microarray analysis demonstrated a decrease in expression of chemokine-encoding genes [17]. Hence, we performed a chemokine profile using PCR array gene analysis of WT and *Etv5^-/-^* Sertoli cells. The results established significant decreases in expression of 14 of the 74 chemokine-related genes analyzed (data not shown). We confirmed by real-time PCR that expression of *Ccl7*, *Ccl9*, *Ccl12*, and *Cxcl5* was decreased ∼50% (Fig. 1A). Expression of five housekeeping genes and other Sertoli genes (Fig. 1B) was unchanged in freshly isolated *Etv5^-/-^*compared with WT Sertoli cells, indicating that decreases observed in chemokine-related mRNA transcripts in *Etv5^-/-^* Sertoli cells were specific, and not related to in vitro manipulation.

### Etv5^-/-^ Sertoli Cells Have Decreased Chemoattractive Ability

**Figure 2:**
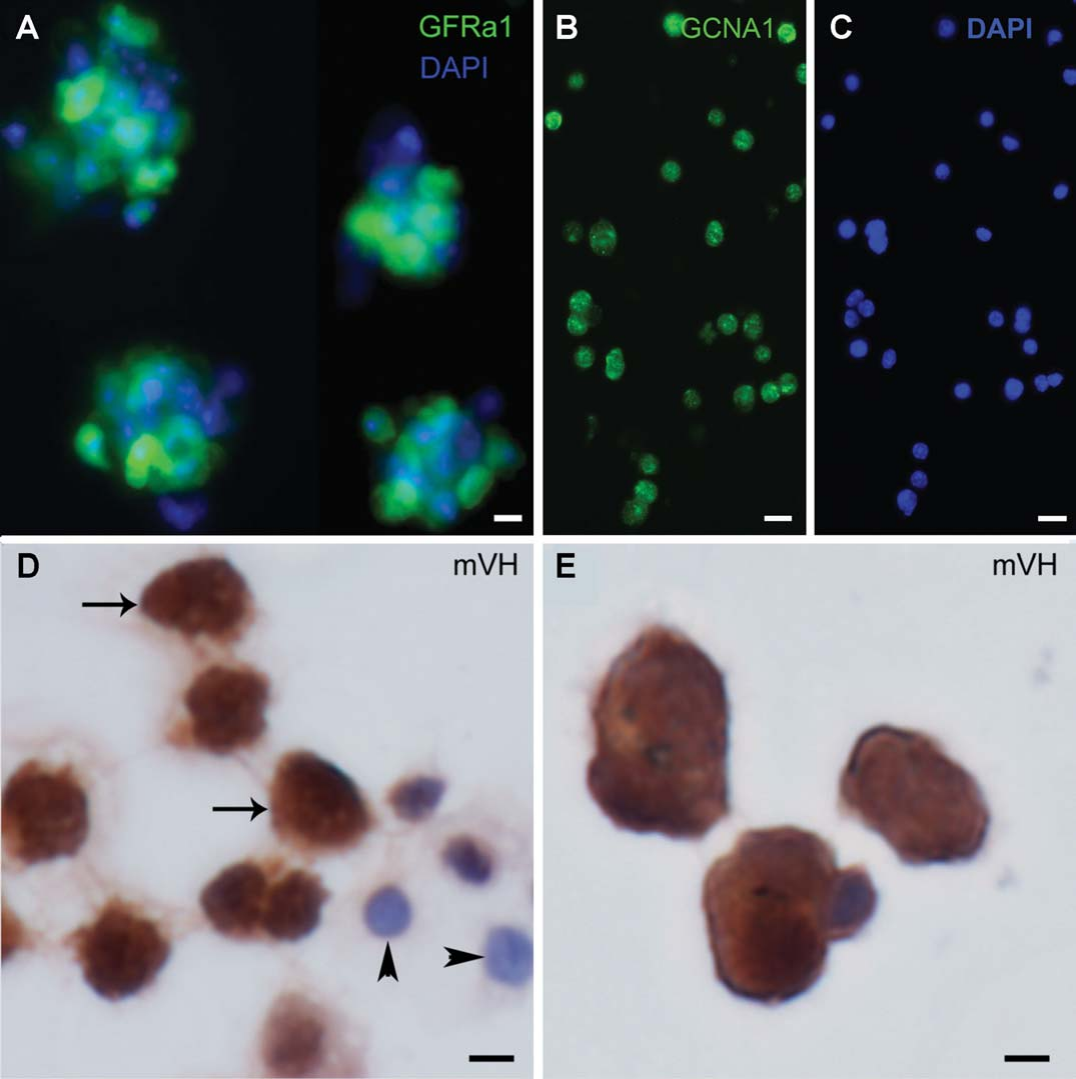
Stem/progenitor spermatogonia and other germ cells isolated by STAPUT. Different germ cell populations were isolated from C57Bl/6 mice at specific ages using the STAPUT method. (A): Type A spermatogonia were further subjected to magnetic-activated cell sorting for enrichment in stem/progenitor spermatogonia. About 80% of these cells show intense immunofluorescence for GFRa1, a spermatogonial stem cell marker (green Alexa Fluor 488 staining). Scale bar = 10 µm. (B): These cells also express GCNA1, a germ cell marker (green Alexa Fluor 488 staining). Scale bar = 25 µm. (C): DAPI staining for cells expressing GCNA1. Scale bar = 25 µm. (D): Immunohistochemistry of type B spermatogonia, leptotene/zygotene spermatocytes (arrows) show that they express mVH, an early germ cell marker, and Sertoli cells (arrowheads) do not express the protein. Scale bar = 10 µm. (E): Early pachytene spermatocytes also express mVH. Scale bar = 10 µm. Abbreviations: DAPI, 4',6-diamidino-2-phenylindole; GFRa1, GDNF familiy membrane receptor alpha 1; GCNA1, germ cell nuclear antigen 1; mVH, mouse vasa homolog.

Stem/progenitor spermatogonia were isolated from 5- to 6-day-old pups using the STAPUT method and MACS purification with GFRa1 antibody as described earlier. About 80% of the isolated cells expressed GFRa1 and all expressed GCNA1, indicating that the germ cell population was enriched for GFRa1 stem/progenitor spermatogonia (Fig. 2A–2C). Type B spermatogonia, L/Z, and P spermatocytes were characterized based on the size and expression of mVH, a germ cell marker (Fig. 2D, 2E). RS were characterized based on the size.

**Figure 3:**
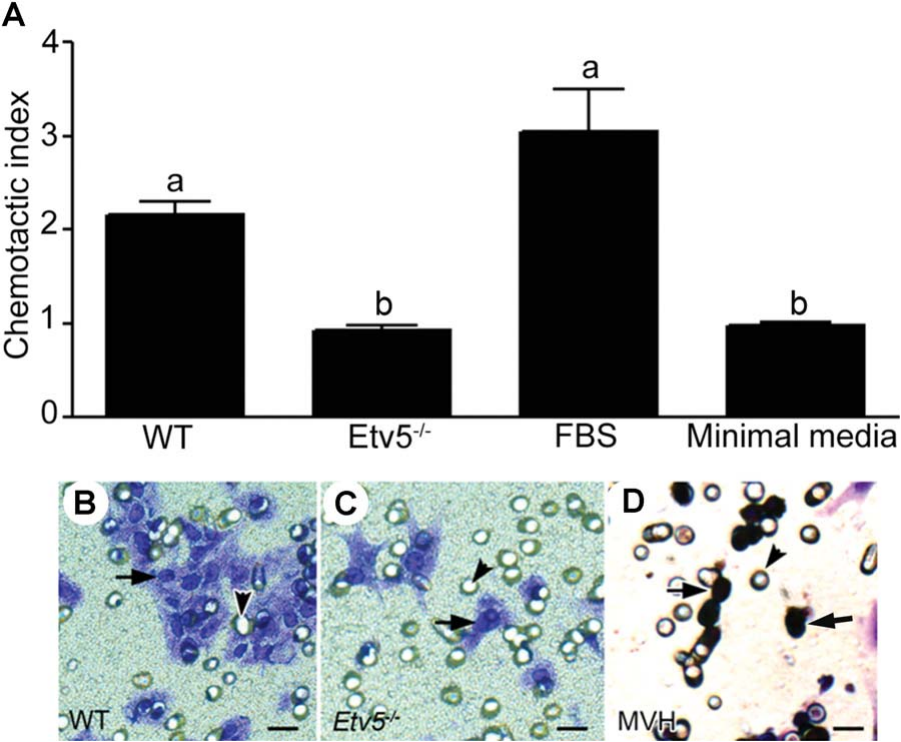
*Etv5^-/-^* Sertoli cells have decreased chemoattractive ability. Stem/progenitor spermatogonia were isolated with the STAPUT/magnetic-activated cell sorting method using a GDNF familiy membrane receptor alpha 1 (GFRa1) antibody. They were used for chemotaxis assays toward Sertoli cells using 8-µm-pore transwell units in 24-well plates. The ability of stem/progenitor spermatogonia to migrate toward Sertoli cells was expressed as their chemotactic index. (A): Results indicate that the chemotactic index of stem/progenitor spermatogonia toward WT Sertoli cells was twice more than that toward *Etv5^-/-^* Sertoli cells. The chemotactic index toward the positive control (10% FBS) was approximately threefold that of the negative control (minimal media). The results are representative of three independent experiments and are expressed as mean ± SEM. Values with different letters were significantly different (*p* < .01). (B–D): The panel shows representative images of migrated cells. (B): Stem/progenitor spermatogonia after migration toward WT Sertoli cells and (C) after migration toward *Etv5^-/-^* Sertoli cells as a comparison. There was a marked decrease in the number of cells that migrated toward *Etv5^-/-^* Sertoli cells. (D): mVH expression of migrated cells showed that more than 95% of the chemoattracted cells were germ cells (arrows). Arrowheads represent the 8 µm pores of the transwell unit. Scale bar = 10 µm. Abbreviations: *Etv5*, Ets variant gene; FBS, fetal bovine serum; mVH, mouse vasa homolog; WT, wild type.

The chemotactic index was defined as the number of germ cells migrating toward Sertoli cells. This index was significantly decreased (over 50%, *p* < .001) when GFRa1^+^ germ cells migrated toward *Etv5^-/-^* Sertoli cells compared with WT Sertoli cells (Fig. 3A–3C). The chemotactic index toward *Etv5^-/-^* Sertoli cells was not statistically different from that toward the negative control, which was minimal media alone. This indicated that *Etv5^-/-^* Sertoli cells had no demonstrable chemoattractive ability for stem/progenitor spermatogonia. The chemotactic index toward the FBS positive control was approximately threefold greater than that toward the negative control. The GFRa1^+^ cells that had migrated also expressed mVH, confirming that these cells were germ cells (Fig. 3D).

### Sertoli Cells Chemoattract Only Stem/Progenitor Spermatogonia and Not Other Germ Cells

**Figure 4:**
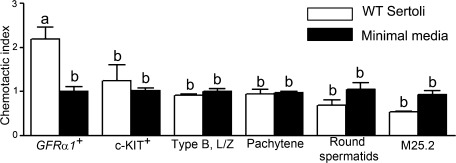
Chemoattractive ability of WT Sertoli cells is specific for stem/progenitor spermatogonia. Chemotaxis assays were performed to assess if Sertoli cells had a specific effect on GFRa1^+^ cells compared with cKIT^+^, type B spermatogonia, leptotene/zygotene spermatocytes, pachytene spermatocytes, or round spermatids. A mesoangioblast stem cell line (M25.2) was also used as a control. The chemotactic index of stem/progenitor spermatogonia (GFRa1^+^) toward WT Sertoli cells was significantly greater than all other cell types. The chemotactic index of germ cell fractions other than GFRa1^+^ cell fraction migrating toward WT Sertoli cells was not significantly different from that migrating toward minimal media alone. The results are representative of three independent experiments and are expressed as mean ± SEM. Values with different letters were significantly different (*p* < .05). Abbreviations: *GFRa1*, GDNF familiy membrane receptor alpha 1; L/Z, leptotene/zygotene spermatocytes; type B, type B spermatogonia; WT, wild type.

The number of GFRa1^+^ germ cells (stem/progenitor spermatogonia) migrating toward WT Sertoli cells was significantly higher (*p* < .01) compared with other germ cell fractions (Fig. 4). The migration of other germ cell fractions, including cKIT^+^ cells (differentiating spermatogonia) toward WT Sertoli cells was not significantly different from migration toward minimal media alone. A mesoangioblast cell line (M25.2, a generous gift from Dr. Suzanne Berry, University of Illinois) was also used as a control and there was no increase in the chemotactic index of these cells toward Sertoli cells (Fig. 4). These results indicate that the chemoattractive ability of Sertoli cells is a specific effect on stem/progenitor spermatogonia only.

### Recombinant CCL9 Restores the Chemoattractive Ability of *Etv5^-/-^* Sertoli Cells

**Figure 5:**
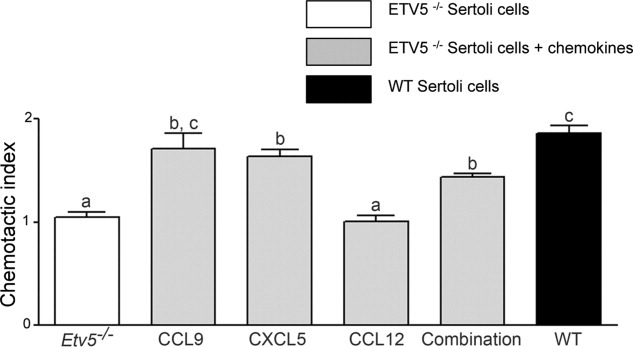
Chemokines increase the chemoattractive ability of *Etv5^-/-^* Sertoli cells. Recombinant proteins CCL9, CXCL5, and CCL12 were added individually or in combination along with *Etv5^-/-^* Sertoli cells. CCL9 and CXCL5 significantly increased the chemoattractive ability of *Etv5^-/-^* Sertoli cells. Addition of CCL9 was able to restore the chemoattractive ability of *Etv5^-/-^* Sertoli cells to WT levels. Adding CCL12 did not increase the chemoattraction of these cells. The combination of all three chemokines significantly increased the chemoattractive ability of *Etv5^-/-^* Sertoli cells, but did not restore it to WT levels. Results are representative of three independent experiments and are expressed as mean ± SEM. Values with different letters were significantly different (*p* < .05). Abbreviations: CCL9, C-C-motif ligand 9; CXCL5, C-X-C motif ligand 5; CCL12, C-C-motif ligand 12; *Etv5*, Ets variant gene; WT, wild type.

As *Ccl9, Ccl12*, and *Cxcl5* were the chemokines showing the greatest (more than 60%) downregulation in *Etv5^-/-^* Sertoli cells, we sought to rescue the decreased ability of *Etv5^-/-^* Sertoli cells to chemoattract stem/progenitor spermatogonia using these chemokines. Recombinant proteins were added at a final concentration of 50 ng/ml, individually or in combination, to *Etv5^-/-^* Sertoli cells in the lower chamber of the transwell units and chemotaxis assays performed. Only recombinant CCL9 was able to rescue the chemoattractive ability of *Etv5^-/-^* Sertoli cells to WT levels as there was no statistically significant difference between the chemotactic index of stem/progenitor spermatogonia toward untreated WT and *Etv5^-/-^* Sertoli cells that were treated with recombinant CCL9 (Fig. 5). Addition of CXCL5 to *Etv5^-/-^* Sertoli cell cultures significantly increased the chemoattractive ability above the *Etv5^-/-^* levels, but did not fully restore it to WT levels. Recombinant CCL12 did not show any effect (Fig. 5). The combination of all three chemokines also significantly increased the chemoattractive ability above *Etv5^-/-^* levels, but did not restore it to that seen with WT Sertoli cells (Fig. 5).

### Expression of CCL9 and CCR1 in the Testis

**Figure 6:**
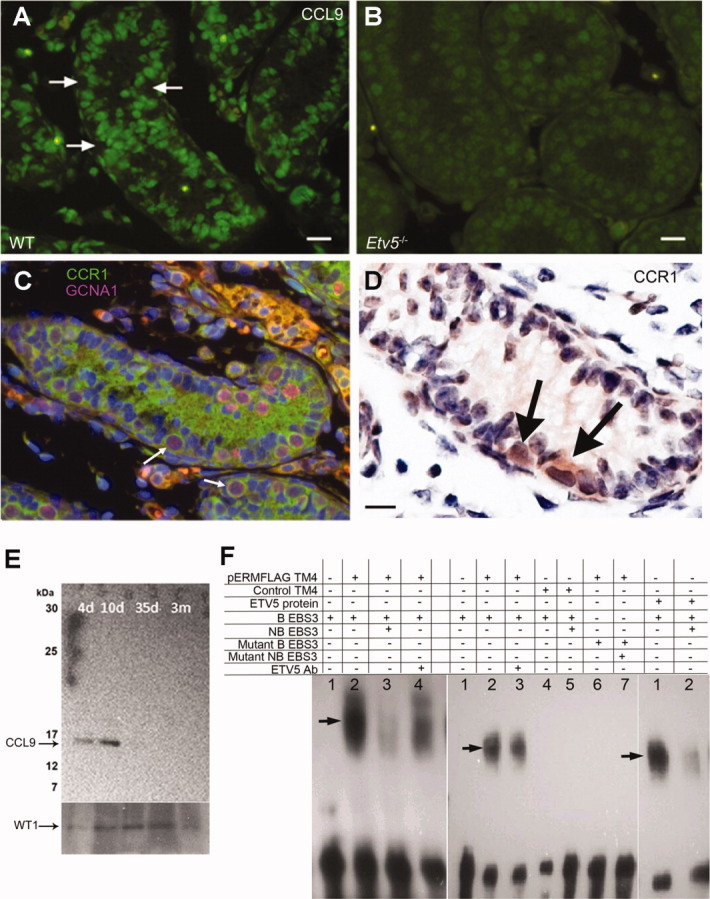
Expression of CCL9 in mouse testis. (A): Immunofluorescence of 8-day WT mouse testis showing strong expression of CCL9 in Sertoli cells (green Alexa Fluor 488 staining). Arrows indicate locations of stem/progenitor spermatogonia, which are negative for CCL9. Scale bar = 25 µm. (B): Immunofluorescence of 8-day *Etv5^-/-^* mouse testis showing a sharp decrease of CCL9 expression in Sertoli cells. Scale bar = 15 µm. (C): Immunofluorescence of 8-day WT mouse testis showing expression of CCR1, the receptor for CCL9, in stem/progenitor spermatogonia. Type A spermatogonia show double-staining for CCR1 (green Alexa Fluor 488 staining) and GCNA1 (pink Alexa Fluor 594 staining). The green staining in the lumen is background staining. Scale bar = 25 µm. (D): Immunohistochemistry of 6-day WT mouse testis showing expression of CCR1 in stem/progenitor spermatogonia (arrows). Scale bar = 15 µm. (E): Western blot of protein extracts from isolated WT seminiferous tubules of 4-, 10-, 35- and 90-day-old mice with anti-CCL9 and anti-WT1 antibodies, showing that CCl9 expression is restricted to early steps of spermatogenesis. (F): Electrophoretic mobility shift assays to investigate ETV5 binding to its consensus sequence in the *Ccl9* promoter. The left panel shows gel shifts using nuclear extracts of TM4 cells after transfection with the pERMFLAG construct (pERMFLAG TM4). 1: biotinylated probe alone (B EBS3); 2: nuclear extract of pERMFLAG TM4 and B EBS3, with apparent gel shift (arrow); 3: nuclear extract of pERMFLAG TM4 with B EBS3 and nonbiotinylated probe (NB EBS3) showing outcompetition; and 4: nuclear extract of pERMFLAG TM4 with B EBS3 and ETV5 antibody (ETV5 Ab) showing a decrease of DNA/protein binding. The middle panel shows gel shifts using again pERMFLAG TM4 nuclear extracts. 1: B EBS3 alone; 2: pERMFLAG TM4 with B EBS3, showing apparent gel shift (arrow); 3: pERMFLAG TM4 with B EBS3 and ETV5 Ab showing decrease of protein-DNA binding; 4: nuclear extract of TM4 cells after transfection with control vector (Control TM4), and incubated with B EBS3; 5: control TM4 with B EBS3 and NB EBS3, 6: pERMFLAG TM4 with mutant B EBS3, and 7: pERMFLAG TM4 with mutant NB EBS3. Gel shifts with NB EBS3 substantially, but not completely outcompeted the protein-DNA complex suggesting the presence of other factors in the complex. There was no shift of protein-DNA complexes with mutant EBS3 sequences or TM4 cells transfected with control vector. The right panel shows gel shifts of purified ETV5 protein using pET vector (ETV5 protein). 1: ETV5 protein with B EBS3, showing gel shift (arrow) and 2: ETV5 protein with B EBS3 and NB EBS3 showing outcompetition. The data confirms a possible ETV5-EBS3 binding. Abbreviations: CCL9, C-C-motif ligand 9; CCR1, C-C-receptor type 1; GCNA, germ cell nuclear antigen 1; *Etv5*, Ets variant gene; ETV5 protein, Ets variant protein; B EBS3, biotinylated Ets binding site 3; NB EBS3, nonbiotinylated Ets binding site 3; WT, wild type.

As CCL9 was the most active chemokine tested, we confirmed its expression in WT Sertoli cells by immunohistochemistry (Fig. 6A) and its downregulation in *Etv5^-/-^* mice (Fig. 6B). The receptor for CCL9, CCR1, was expressed in gonocytes, and later in undifferentiated spermatogonia, but not in Sertoli cells (Fig. 6C, 6D). This pattern of expression was confirmed by real-time PCR (data not shown). Western blot analysis of WT Sertoli cell extracts indicated that these cells expressed CCL9 and that there was a downregulation of CCL9 protein expression starting 10 days after birth with no expression of the protein after 35 days (Fig. 6E). Therefore, a functional interaction between CCL9 and CCR1 in the testis is likely, but might be restricted to the proliferative period of Sertoli cells.

### ETV5 Interaction with the *Ccl9* Promoter

To assess if there was interaction between ETV5 and the C*cl9* promoter, EMSAs were performed. The Sertoli cell line TM4 was transfected with pErmFLAG [42], which resulted in a 10,000-fold increase of mRNA expression of *Etv5* compared with nontransfected controls. Nuclear extracts prepared from these cells were incubated with three biotinylated putative ETV5-binding site sequences (EBS1-3) in the *Ccl9* promoter region. Gel shifts were observed when nuclear extracts were combined with biotinylated EBS3 sequences (B EBS3), indicating protein-DNA interaction (Fig. 6F). A 200-fold excess of nonbiotinylated EBS3 probes (NB EBS3) substantially, but not completely, outcompeted protein-DNA binding, suggesting that other factors might be present in the complex. Incubation with ETV5 antibody seemed to alter protein-DNA binding, rather than producing a consistent supershift, which is in agreement with the data of Lin et al. [42]. Shifts of protein-DNA complexes were not observed when nuclear extracts from TM4 cells transfected with the control vector were incubated with B EBS3 or when mutant EBS3 sequences were incubated with cells transfected with the *Etv5* construct (Fig. 6F). Purified ETV5 protein also produced a gel shift that was almost completely outcompeted with the NB EBS3 probe, confirming the data obtained with nuclear extracts. Taken together, these results indicate that ETV5 is a good candidate regulator of CCL9 expression.

### Downregulation of *Ccr1* Induces a Reduction in the Migration of Stem/Progenitor Spermatogonia

**Figure 7:**
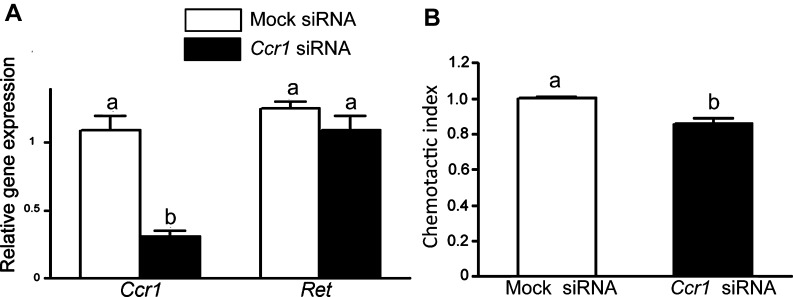
RNA interference of *Ccr1* in stem/progenitor spermatogonia. (A): RNA interference of *Ccr1* in stem/progenitor spermatogonia downregulated the expression of the gene 70% compared with mock siRNA-treated stem/progenitor spermatogonia. Expression of *Ret* in *Ccr1* siRNA transfected stem progenitor/spermatogonia showed that the gene was not affected due to transfection. (B): Chemotaxis assays were performed with stem/progenitor spermatogonia that were transfected with *Ccr1* and mock siRNA for 24 hours. The chemotactic index of transfected cells toward wild-type Sertoli cells was 0.86 and 1.002, indicating a 14% reduction in the migration of these cells. Results are representative of three independent biological repeats for RNA interference and two technical repeats for cell migration assays with the stem/progenitor spermatogonia transfected with *Ccr1* siRNA. Values are expressed as mean ± SEM. Values with different letters were significantly different (*p* < .05).

*Ccr1* was downregulated in primary stem/progenitor spermatogonia ∼70% using RNA interference (Fig. 7A). Stem/progenitor spermatogonia downregulated for *Ccr1* were subjected to chemotaxis assays. There was a 14% reduction in the chemotactic index of these cells toward WT Sertoli cells, which was statistically significant (Fig. 7B). Although there was a reduction in migration of these cells, compensatory mechanisms by other chemokines or receptors probably limited this effect.

## DISCUSSION

It has been recently demonstrated that the absence of ETV5 in SSCs decreases their proliferation [19, 21] and contributes to the decline in their numbers [20]. However, decreased proliferation would reduce, but not eliminate stem/progenitor spermatogonia in the seminiferous tubules, and the critical question is how the loss of ETV5 ultimately results in a total loss of stem/progenitor spermatogonia.

The present study sought to identify and determine the functional significance of chemokines produced by Sertoli cells that are regulated by ETV5. Our results indicate that downregulation of specific chemokines such as CCL9 and CXCL5 in *Etv5^-/-^* Sertoli cells leads to impairments in the ability of these Sertoli cells to chemoattract stem/progenitor spermatogonia. This may be a key aspect of the mechanism by which loss of ETV5 protein leads to a progressive loss of germ cells in the *Etv5^-/-^* testis. Downregulation of chemokines is specific, because the expression of other genes is not affected in freshly isolated *Etv5^-/-^* Sertoli cells. If a loss of chemokine expression had occurred as a result of subsequent culture conditions, and not of gene ablation, then both WT and *Etv5^-/-^* Sertoli cells would have been affected, without changes in differential expression.

The stem cell niche plays an essential role in maintaining the balance between self-renewal and differentiation of stem cells. As stem/progenitor spermatogonia have direct cell-to-cell contact only with Sertoli cells in the seminiferous tubules, and Sertoli cells are the major source of ETV5 in the postnatal and adult testis [5, 17], changes in *Etv5^-/-^* Sertoli cells are likely involved in the loss of germ cells in these knockout mice. The loss of stem/progenitor spermatogonia in the *Etv5^-/-^* testes starts between day 4 and day 8 after birth [20], and by day 16 the testis weight is already 35% smaller than WT [20]. In the WT testis, CCL9 is expressed at least until day 10, if not longer (but not after day 35; Fig. 6E). Therefore, it is plausible that lack of CCL9 in Etv5^-/-^ mice might impair stem/progenitor spermatogonia maintenance and migration shortly after birth. In mice, proliferation of Sertoli cells (creating new niches) occurs until day 16. As CCL9 is present during this period, and that we demonstrate migration using spermatogonia and Sertoli cells aged 5–8 days, it is possible that chemotaxis ensures stem/progenitor spermatogonia filling the niches [5]. A reduction in the migration of spermatogonia to fill new niches formed by proliferating Sertoli cells might result in Sertoli cell only regions, as observed in the *Etv5^-/-^* testis, and could contribute to the overall decline in testis weight. Similarly, the lack of colonization of *Etv5*^-/-^ seminiferous tubules by WT stem/progenitor spermatogonia [18] could be attributed, at least in part, to a lack of proper chemokine expression.

Previous literature indicates that chemokine signaling may be involved in maintaining stem cells and progenitors in other niches. For example, chemokines secreted by the hematopoietic stem cell (HSC) niche are putative regulatory factors of HSCs [24, 43]. The aorta-gonad-mesonephros region generates HSCs and serves as the HSC microenvironment. In particular, in this system chemokines such as CCL9 and CCL3 are produced by the cells comprising the niche, and these chemokines regulate and enhance the repopulating ability of HSCs in vitro [24, 44]. Consistent with a role of niche cells signaling through chemokines to stem cells, neural stem cells (NSCs) express CXCR4, the cognate receptor for the chemokine CXCL12. CXCL12 treatment of quiescent NSCs enhances proliferation, promotes chain migration, and activates intracellular molecular pathways mediating recruitment of these cells toward an injury [25]. Therefore, chemokines and their receptors might play a similar role in spermatogenesis as they do in hematopoiesis and neurogenesis, where they regulate homing/retention of stem cells in the surrounding microenvironment and provide signals for cell growth and differentiation.

Although Sertoli cell chemoattraction or a role of Sertoli cell chemokines in the postnatal testis has not been previously demonstrated, the chemokine CXCL12 is essential for migration of PGCs to the gonadal ridges, and its receptor, CXCR4, is expressed by these cells [26]. PGC and gonocyte migration is not affected in *Etv5^-/-^* mice as stem/progenitor numbers were comparable in neonatal *Etv5^-/-^* and WT mice [20]. This is consistent with our present results that CXCL12 was not among the chemokines decreased in *Etv5^-/-^* testes.

Chemotaxis assays were performed to study the functional role of chemokines on stem/progenitor spermatogonia. These studies clearly indicated that Sertoli cells were chemoattractive for GFRa1^+^ stem/progenitor spermatogonia, the first demonstration that Sertoli cells have a chemotactic effect on these cells and that other germ cell populations such as type B spermatogonia, L/Z, P spermatocytes, and RS were not chemoattracted by Sertoli cells. In addition, the chemoattractive ability of Sertoli cells was significantly decreased in *Etv5^-/-^* mice, indicated by a reduced number of stem/progenitor spermatogonia migrating toward *Etv5^-/-^* Sertoli cells compared with their age-matched WT controls.

Our results demonstrate that two of the three chemokines identified as being reduced in *Etv5^-/-^* Sertoli cells were chemoattractive for stem/progenitor spermatogonia (CCL9 and CXCL5). However, our results also indicated that adding a cocktail of the three recombinant chemokines (CCL9, CCL12, and CXCL5) to *Etv5^-/-^* Sertoli cells did not restore their chemoattractive ability to WT levels. Therefore, the cell migratory behavior might vary depending on the concentration or the combination of the chemoattractants used and the combination of chemokines may synergistically increase or decrease the migratory behavior of stem cells [45]. In the present study, CCL12 did not increase the chemoattractive ability of *Etv5^-/-^* Sertoli cells, suggesting that this chemokine does not have a chemotactic role postnatally. The inability of the combination of all chemokines to fully restore values of *Etv5^-/-^* Sertoli cells back to WT levels, indicates that there might be changes in *Etv5^-/-^* Sertoli cells other than reduction in the chemokines examined that affected chemoattraction. These are likely other growth factors, adhesion molecules, and/or proteolytic enzymes produced by Sertoli cells that have a role in the complex interactions between Sertoli cells and stem/progenitor spermatogonia. The behavior and activity of chemokines is also controlled in a complex manner by several mechanisms that include synergistic interactions, glycosaminoglycans binding properties, and interactions with accessory molecules [46]. These factors, absent in our culture conditions, might influence their mobilization, transport, and rate of degradation, thereby determining the sites and duration of their action.

As CCL9 was sharply reduced in *Etv5^-/-^* Sertoli cells and attracted stem/progenitor spermatogonia at the highest level in vitro, we further studied the expression patterns of CCL9 and its receptor CCR1 in the testis. Immunohistochemical localization of CCL9 confirmed its expression in Sertoli cells, consistent with our PCR-array and real-time PCR data. Similarly, CCL7 and its receptor, which are also expressed in Sertoli cells and spermatogonia, might also function in the maintenance of stem/progenitor spermatogonia within the niche [1]. Though there was strong CCL9 protein expression in seminiferous tubules neonatally, it decreased to undetectable levels between day 10 and day 35, supporting the hypothesis that proliferating Sertoli cells create new niches and stem/progenitor spermatogonia migrate along the basement membrane to occupy them [5]. Furthermore, our EMSA data indicated that ETV5 is a good candidate regulator of *Ccl9* expression. EMSAs performed with different concentrations of NB probes or different ETV5 antibody concentrations will clarify in the future whether other factors are present in the protein-DNA complex, and the degree of specificity of the interaction.

As expected, the receptor for CCL9, CCR1, was expressed by undifferentiated spermatogonia. Microarray analysis of Thy1^+^ testis cell populations also showed that expression of *Ccr1* and many other chemokine receptors was increased more than 10-fold in these cells [47] suggesting the importance of chemokine signaling in maintenance of stem/progenitor spermatogonia in their microenvironment. In the chemotaxis assays performed with *Ccr1* siRNA spermatogonia, there was only a 14% reduction in the chemotactic index suggesting a redundancy of chemokine signaling by either the receptors or the ligands. Although chemokines and their receptors have been shown to be crucial for stem cell homing and maintenance in the hematopoietic and neural systems, targeted deletions of their genes usually only led to mild phenotypic defects [48–50]. Similarly, *Ccr1^-/-^* mice are fertile and have very minor phenotypes in other organs [49] suggesting a compensatory mechanism in chemokine signaling. Hence, transplantation studies of germ cells that are knocked out for a single chemokine receptor will not give an insight into the complex signaling mechanism of chemokines produced by Sertoli cells in maintaining stem/progenitor spermatogonia in their microenvironment.

## CONCLUSION

Our data show for the first time that Sertoli cells are chemoattractive for stem/progenitor spermatogonia in the prepubertal testis. We demonstrated that ETV5 is responsible for this process, possibly by regulating the production of chemokines such as CCL9 by Sertoli cells (supporting information Figure S1). However, our data also indicate that more than one chemokine is involved. A decrease in chemokine production by *Etv5^-/-^* Sertoli cells, and the resulting decline of their chemotactic activity reduce the migration of stem/progenitor spermatogonia to newly formed Sertoli cells/niches. This helps to explain the loss of germ cells in *Etv5*^-/-^ mice.
